# The Effects of Serum Proteins on Magnesium Alloy Degradation *in Vitro*

**DOI:** 10.1038/s41598-017-14479-6

**Published:** 2017-10-30

**Authors:** Ian Johnson, Wensen Jiang, Huinan Liu

**Affiliations:** 1 Department of Bioengineering, University of California at Riverside, Riverside, CA 92521 USA; 2 Materials Science and Engineering, University of California at Riverside, Riverside, CA 92521 USA

## Abstract

Magnesium (Mg) alloys are promising materials for biodegradable implants, but their clinical translation requires improved control over their degradation rates. Proteins may be a major contributing factor to Mg alloy degradation, but are not yet fully understood. This article reports the effects of fetal bovine serum (FBS), a physiologically relevant mixture of proteins, on Mg and Mg alloy degradation. FBS had little impact on mass loss of pure Mg during immersion degradation, regardless of whether or not a native oxide layer was present on the sample surface. FBS reduced the mass loss of Mg-Yttrium (MgY) alloy with an oxidized surface during immersion degradation, but increased the mass loss for the same alloy with a metallic surface (surface oxides were removed). FBS also influenced the mode of degradation by limiting the depth of pit formation during degradation processes on commercially pure Mg with metallic or oxidized surfaces and on MgY alloy with oxidized surfaces. The results demonstrated that serum proteins had significant interactions with Mg-based biodegradable metals, and these interactions may be modified by alloy composition and processing. Therefore, proteins should be taken into account when designing experiments to assess degradation of Mg-based implants.

## Introduction

Magnesium (Mg) alloys are promising materials for biodegradable medical implants because of their desirable mechanical and biological properties^1–4^. The clinical translation of Mg alloys requires improved control over their degradation rates in the human body. However, significant differences in Mg alloy degradation measured *in vitro* versus *in vivo* have been reported^5,6^, and these differences can affect the efficacy of rapid screening *in vitro* for clinical translation of Mg alloys. Physiological fluids are rich in aggressive ions that promote the degradation of Mg and its alloys^7,8^. Proteins are another major component in physiological fluids, but their effects on Mg degradation are still poorly understood. Therefore, the objective of this study is to investigate the role of proteins in Mg alloy degradation *in vitro*, and thus improve our ability to bridge the gap between *in vitro* and *in vivo* studies of Mg-based implants.

Proteins can promote or inhibit Mg degradation depending on many different factors, and their effects on metal degradation are often contrary in the literature. Proteins adsorb to alloys through electrostatic or hydrophobic interactions^9^. Surface roughness^10^ and other physical or chemical properties^11^ influence protein adsorption; some surfaces can adsorb one type of protein preferentially over another type of protein^10,11^. The specific effects of proteins on metal degradation depend on the alloy composition, alloy surface, and the types of proteins involved.

This study focuses on investigating the complex interactions between proteins and Mg surfaces to enable the development of strategies for controlling Mg degradation in the body, which is critical for clinical translation of Mg alloys. Fetal bovine serum (FBS) was used to represent the proteins in the physiological environment, because it contains most of the proteins in blood that will come into direct contact with medical implants. Commercially pure Mg and Mg-Yttrium (Y) binary alloys with either oxidized or metallic surfaces were included to represent the effects of bulk composition and surface condition on the degradation of Mg-based biomaterials in the presence or absence of proteins, thus enabling a comprehensive examination of the roles that serum proteins could play on the degradation of Mg-based biomaterials.

## Materials and Methods

### Preparation of Magnesium-based Samples

The as-rolled 250 µm-thick pure magnesium foil (Goodfellow Corporation, as-rolled, 99.9% purity) had a thermal oxide layer on its surface and was called cpMg_O in this study with “cp” indicating commercially pure and “O” indicating the presence of oxides on the surface. Some of the cpMg_O samples were grinded using 600, 800, and 1200 grit silicon carbide abrasive papers (PACE Technologies) sequentially to remove the oxidized layer on the surface, and were referred to as cpMg in this study. The term cpMg* was used to refer to both cpMg_O and cpMg in this article.

Magnesium-4 wt.% yttrium alloy was prepared by melting magnesium with 4 wt.% yttrium (Y) in an argon (Ar) protected environment and casting as an ingot. The as-cast magnesium-yttrium alloy ingot was cut into 250 µm-thick discs using a wire electric discharge machine (AgieCharmilles, Agiecut 200 VHP). The as-produced alloy discs had a thermal oxide layer on their surface and were called MgY_O in this study. Some of the MgY_O samples were grinded using 600, 800, and 1200 grit silicon carbide abrasive papers sequentially to remove the oxidized layer on the surface, and were referred to as MgY in this study. The expression MgY* was used to refer to both MgY_O and MgY in this article.

All of the cpMg* and MgY* samples in this study were cut into dimensions of 10 × 10 mm, ultrasonically cleaned in isopropanol (Sigma-Aldrich, CAS number 67-63-0), and weighed. Both sides of the samples were disinfected under ultraviolet (UV) radiation for at least 1 hour before *in vitro* degradation experiments.

### Immersion Degradation of Magnesium-based Samples

Degradation of cpMg*and MgY* was investigated using the immersion method, adapted from the previously established procedure^12^. Briefly, the respective cpMg* and MgY* samples were immersed in either Dulbecco’s Modified Eagle’s Medium (DMEM) with fetal bovine serum (FBS) or DMEM without FBS under the standard cell culture conditions (37 °C, 5% CO_2_/95% air, humidified, sterile environment) for direct comparison. DMEM supplemented with 10 vol % FBS and 1 vol % penicillin/streptomycin (P/S) is referred to as “DMEM + FBS”, and DMEM without supplements is referred to as “DMEM” in this article. Each sample was immersed in 3 mL of respective immersion solution. DMEM was used as the immersion media because it contained physiologically relevant ions. Complete DMEM (cDMEM) with protein supplements (*i.e*. FBS) has been widely used for *in vitro* cell culture and resembled *in vivo* conditions.

Each cpMg* and MgY* sample was incubated in the respective immersion solution according to prescribed periods. At the beginning of the immersion experiment, the incubation period was shorter and the sample was examined more frequently (1, 2, 4, 8, 16, 24, 48 hours) to provide a higher time resolution for tracking the initial rapid changes of sample mass and pH of immersion solution. Furthermore, the initial period of degradation plays a critical role in the fate of the cells and tissues surrounding the implanted materials. After 3 days of immersion, the incubation period was prolonged to 48 hours to mimic the physiological conditions, and the sample was examined at the end of each incubation period. When the prescribed incubation period ended, the sample was collected from its respective immersion solution and dried in an isotemp oven at 37 °C for 12 hours, or until the sample reached a constant mass. Degradation products that precipitated on the surface of the cpMg* and MgY* samples were left intact, while soluble degradation products remained in the immersion solution. The pH meter was first calibrated with known standards, and was then used to measure the pH of the immersion solution at the end of every prescribed incubation period. The sample was dried, weighed, photographed, disinfected under UV radiation, and then placed in the respective fresh immersion solution for the next incubation period. The same procedure was repeated for each prescribed incubation period. When the sample mass was reduced to less than 3 mg, they became too small to handle and were thus considered as completely degraded at the next incubation period. The mass of each sample after each incubation period (M_i_) was divided by its initial mass (M_0_) to obtain the normalized mass change (M_i_/M_0_). The immersion degradation tests were performed in triplicate for each sample type in the respective immersion solution.

### Potentiodynamic Polarization Measurements for Magnesium-based Samples

The potentiodynamic testing was performed on cpMg* and MgY* at 37 °C before and after immersion in DMEM with or without FBS for 24 hours, according to ASTM standard G 102-89, and repeated for triplicates of each sample. The post-immersion samples were tested in fresh solution of the same type (either with or without FBS) that they were originally immersed in.

Potentiodynamic polarization curves were generated using a Potentiostat/galvanostat (model 273 A; EG&G Princeton applied research). Each sample was connected to the working electrode and immersed in DMEM with or without FBS. An Ag/AgCl reference electrode (part # CHI111, CHI Instruments) and a Pt counter electrode (part # CHI 115, CHI Instruments) were used and immersed in the same solution. The potentiodynamic polarization test was performed at a potential that ranged from −3 V to +1 V, with a 10 mV step size and a 0.5-second step time at a 100 mV/s scan rate. To determine the corrosion current (I_corr_) and corrosion potential (E_corr_), two tangent lines were drawn along the linear portion of the potentiodynamic polarization curves and the intersection of the tangent lines was used to extrapolate I_corr_ and E_corr_ according to the Tafel method.

### Surface Characterization of Magnesium-based Samples

The samples were imaged before and after 24-hours immersion using scanning electron microscopy (SEM; Nova NanoSEM 450; FEI) at a 2500x original magnification with a 15 kV accelerating voltage. The surface elemental composition of the samples was measured using energy dispersive x-ray spectroscopy (EDX) at a 2500x original magnification with a 15 kV accelerating voltage.

### Statistical Analysis of Magnesium Degradation

The R program was used to perform all statistical analysis of the data. The Shapiro-Wilks test was used to test the data for normal distribution. The Bartlett test was used to test the data for homogeneity of variance. The data did not have normal distribution or homogenous variance. A Wild Bootstrap test was used for statistical analysis because the assumptions of data distribution and homogeneous variance are not required. The alloy composition, surface condition, FBS in the immersion media, time, and all possible combinations of these factors were statistically tested for their effects on the changes of sample mass, immersion media pH, E_corr_, and I_corr_. Statistical analysis of the pH only included up to 216-hour time points to ensure equal sample size for statistical testing, because some samples completely degraded after that time point. The presence of FBS in the immersion media was also statistically tested for its effects on the elemental composition of the sample surfaces after degradation. A Bonferroni correction was used to control the familywise error rate with the Wild Bootstrap.

## Results

### The Effects of Degradation on Mass Loss and Solution pH

Alloy composition (cpMg versus MgY) (*p* = 7.99 × 10^−5^), alloy surface type (oxidized versus metallic) (*p* = 7.99 × 10^−5^), presence of proteins (*i.e*. FBS) in the immersion solution (*p* = 3.59 × 10^−4^), and all of their interactions with each other had statistically significant effects on sample mass change during the immersion degradation (Table 1). Immersion time also had a statistically significant effect on sample mass, and it had a statistically significant interaction with alloy composition but not with other factors. None of these factors had a statistically significant effect on immersion media pH, possibly because of the buffering capacity of DMEM.

**Table 1:** Statistical significance of alloy composition, surface type, protein content in immersion media, and immersion time on cpMg* and MgY* degradation; as determined by wild bootstrap. The mass change, pH, I_corr_, and E_corr_ were used as the parameters to represent Mg degradation. The Bonferroni correction resulted in an adjusted α significance value of 3.33 × 10^−3^. The *p* values lower than the adjusted threshold were highlighted in bold, indicating they are statistically significant.

Parameter	Bonferroni Adjusted α = 3.33 × 10^−3^			
	*p* Value			
	M_i_/M_0_	pH	I_corr_	E_corr_
Alloy	**7.99 × 10** ^**−5**^	0.63	**3.99 × 10** ^**−4**^	0.01
Surface	**7.99 × 10** ^**−5**^	0.49	4.75 × 10^−3^	0.4
Protein	**3.59 × 10** ^**−4**^	0.31	0.11	0.94
Alloy:Surface	**7.99 × 10** ^**−5**^	0.07	**1.19 × 10** ^**−4**^	0.07
Alloy:Protein	**7.99 × 10** ^**−5**^	0.93	**2.39 × 10** ^**−4**^	0.83
Surface:Protein	**7.99 × 10** ^**−5**^	0.56	**7.99 × 10** ^**−5**^	0.83
Alloy:Surface:Protein	**7.99 × 10** ^**−5**^	0.98	0.05	0.22
Time	**7.99 × 10** ^**−5**^	0.72	**7.99 × 10** ^**−5**^	**7.99 × 10** ^**−5**^
Alloy:Time	**7.99 × 10** ^**−5**^	0.64	**1.55 × 10** ^**−3**^	0.7
Surface:Time	9.99 × 10^−3^	0.81	0.04	0.17
Protein:Time	0.57	0.91	0.38	0.71
Alloy:Surface:Time	0.03	0.62	**1.19 × 10** ^**−4**^	0.61
Alloy:Protein:Time	0.18	0.46	**3.59 × 10** ^**−4**^	0.37
Surface:Protein:Time	0.83	0.94	**7.99 × 10** ^**−5**^	0.4
Alloy:Surface:Protein:Time	0.29	0.83	0.16	0.99

**Figure 1:**
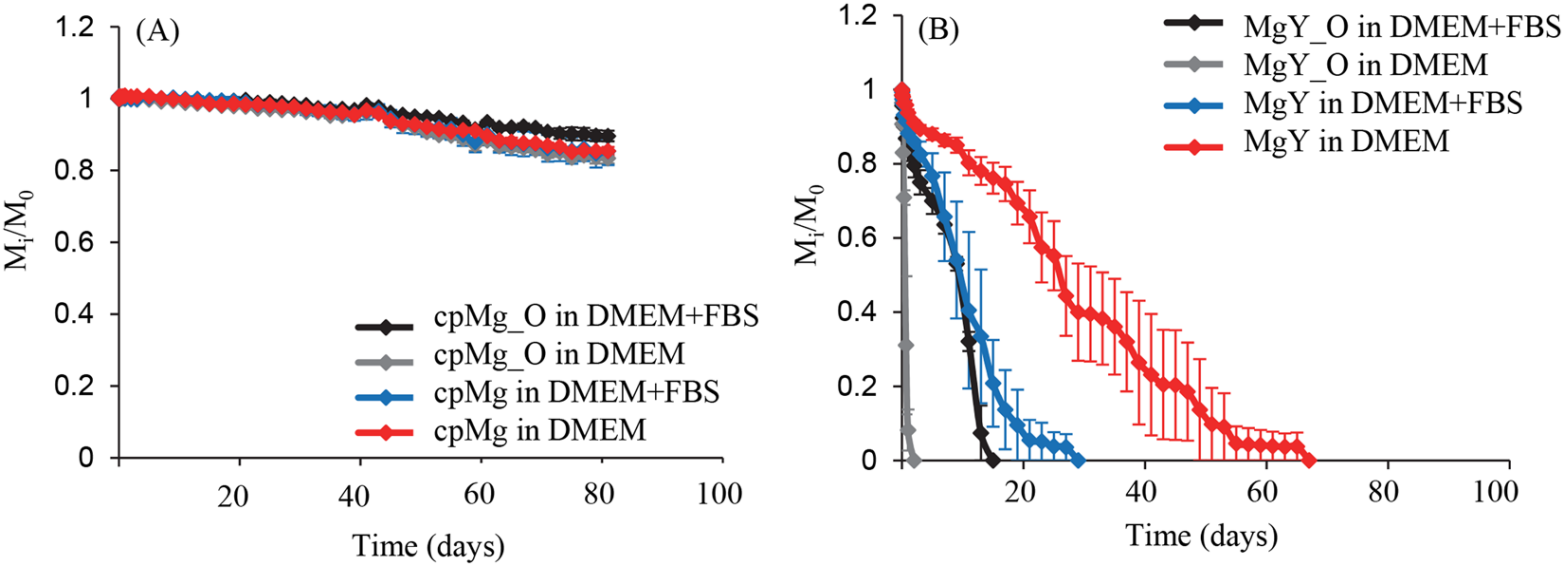
Mass change of (**A**) cpMg* and (**B**) MgY* during immersion in DMEM with and without FBS. Error bars represent standard error.

The cpMg* samples slowly lost mass over time in DMEM with or without FBS (Fig. 1A). The presence of FBS had negligible effect on the mass loss of cpMg* initially, and after 40 days the presence of FBS slightly reduced the mass loss of cpMg_O but did not have much effect on the mass loss of cpMg. MgY* lost mass much more rapidly than cpMg* (Fig. 1B), and the mass loss of MgY* was affected significantly by the presence of FBS. The presence of FBS decreased MgY_O mass loss and increased MgY mass loss. MgY_O lost mass more rapidly than MgY regardless of whether or not FBS was present in the immersion solution.

**Figure 2:**
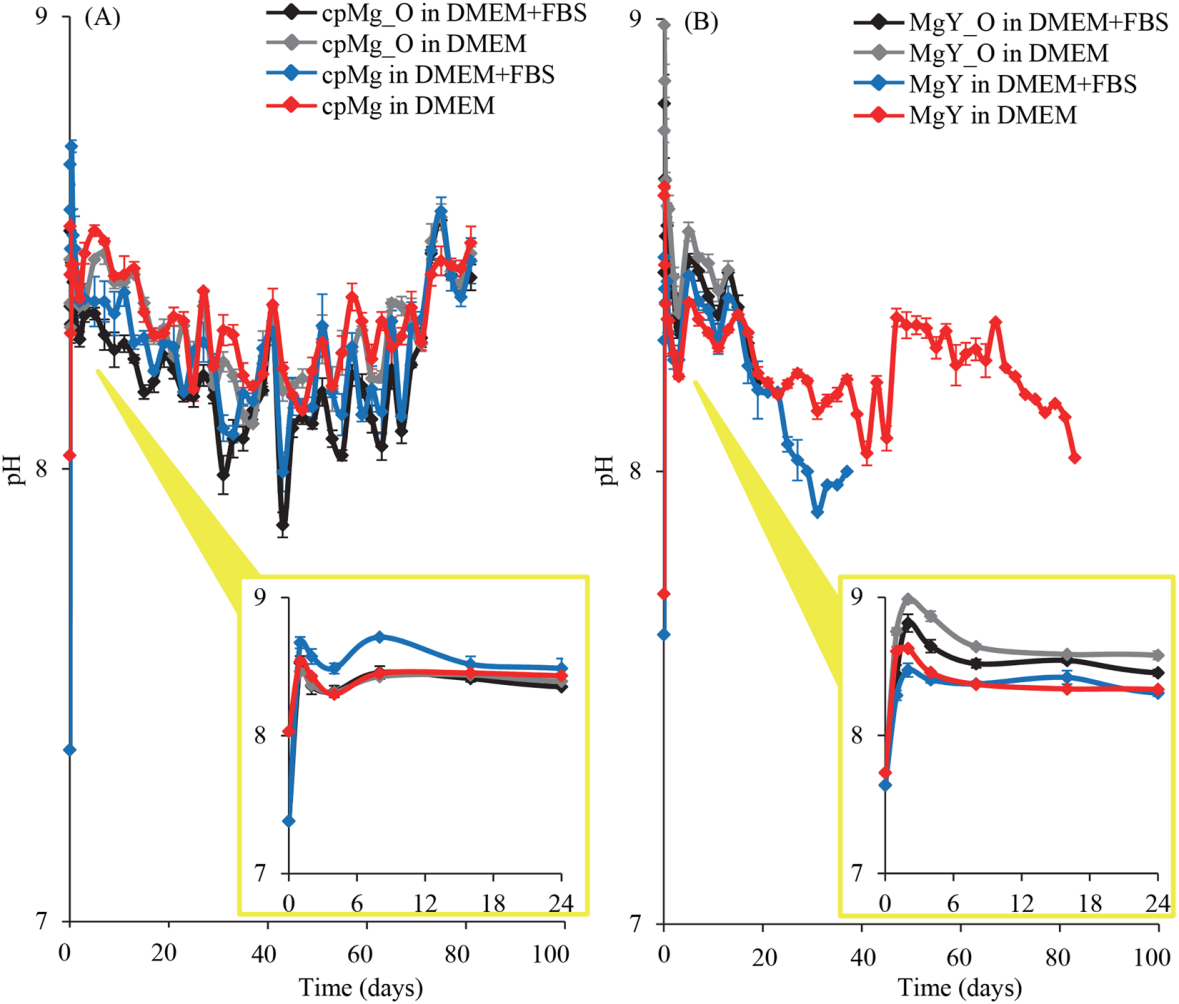
The change in pH of immersion media containing (**A**) cpMg* and (**B**) MgY*. Error bars represent standard error. The insets show the magnified view for the first 24 hours.

Immersion of cpMg* and MgY* increased the pH of the immersion solution (Fig. 2). The pH rapidly increased up to 8.67 after 1 hour of immersion of cpMg*. FBS had negligible effects on the pH changes during immersion of cpMg*. FBS had a slightly more noticeable effect on the pH changes caused by MgY* immersion; and MgY* had a slightly larger spike to its early pH at 1 or 2 hours when FBS was not present.

### The Effects of Degradation on Surface Morphology and Integrity

**Figure 3:**
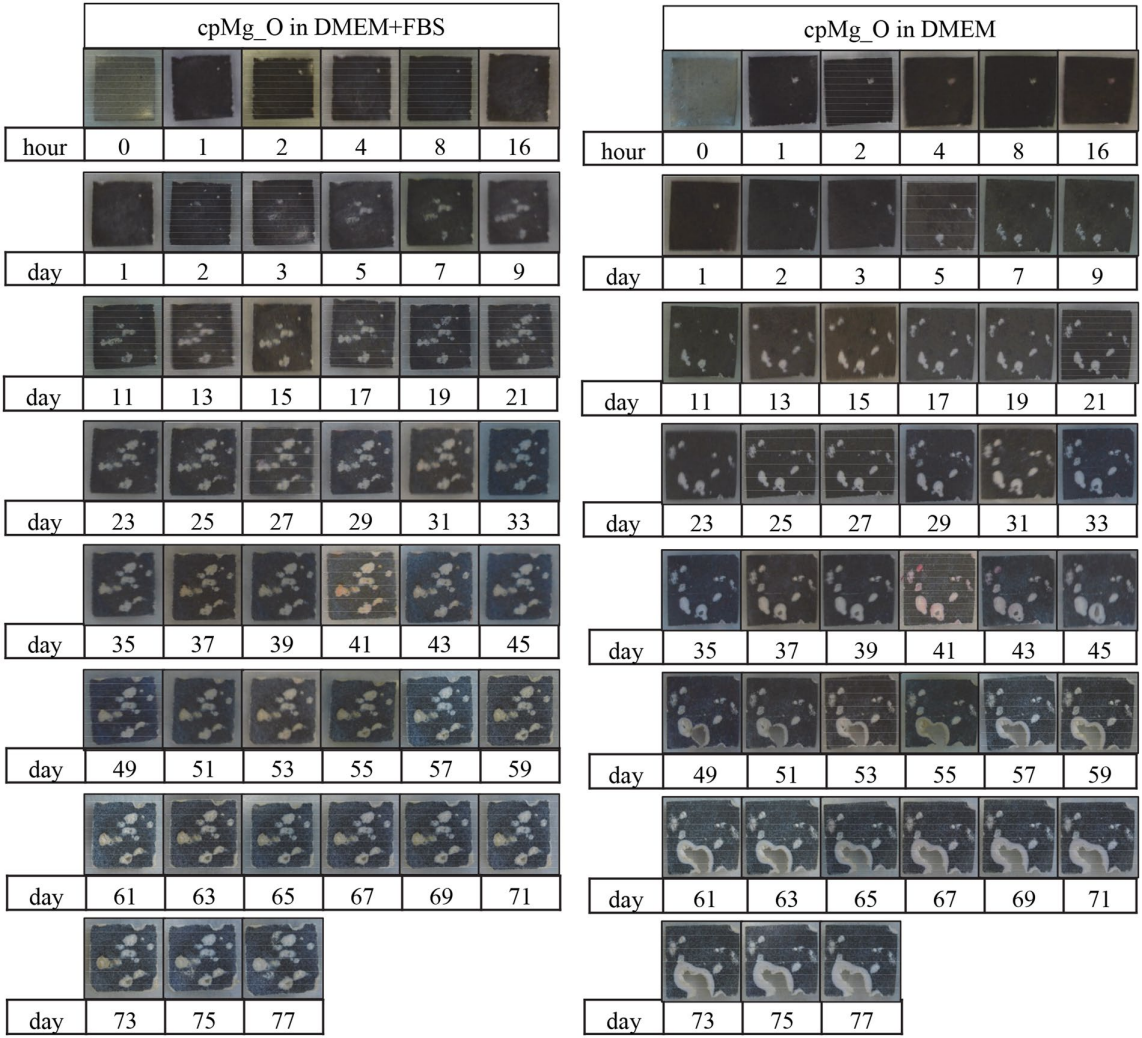
Photographs of cpMg_O showing its macroscopic appearance during immersion degradation in DMEM with and without FBS.

**Figure 4:**
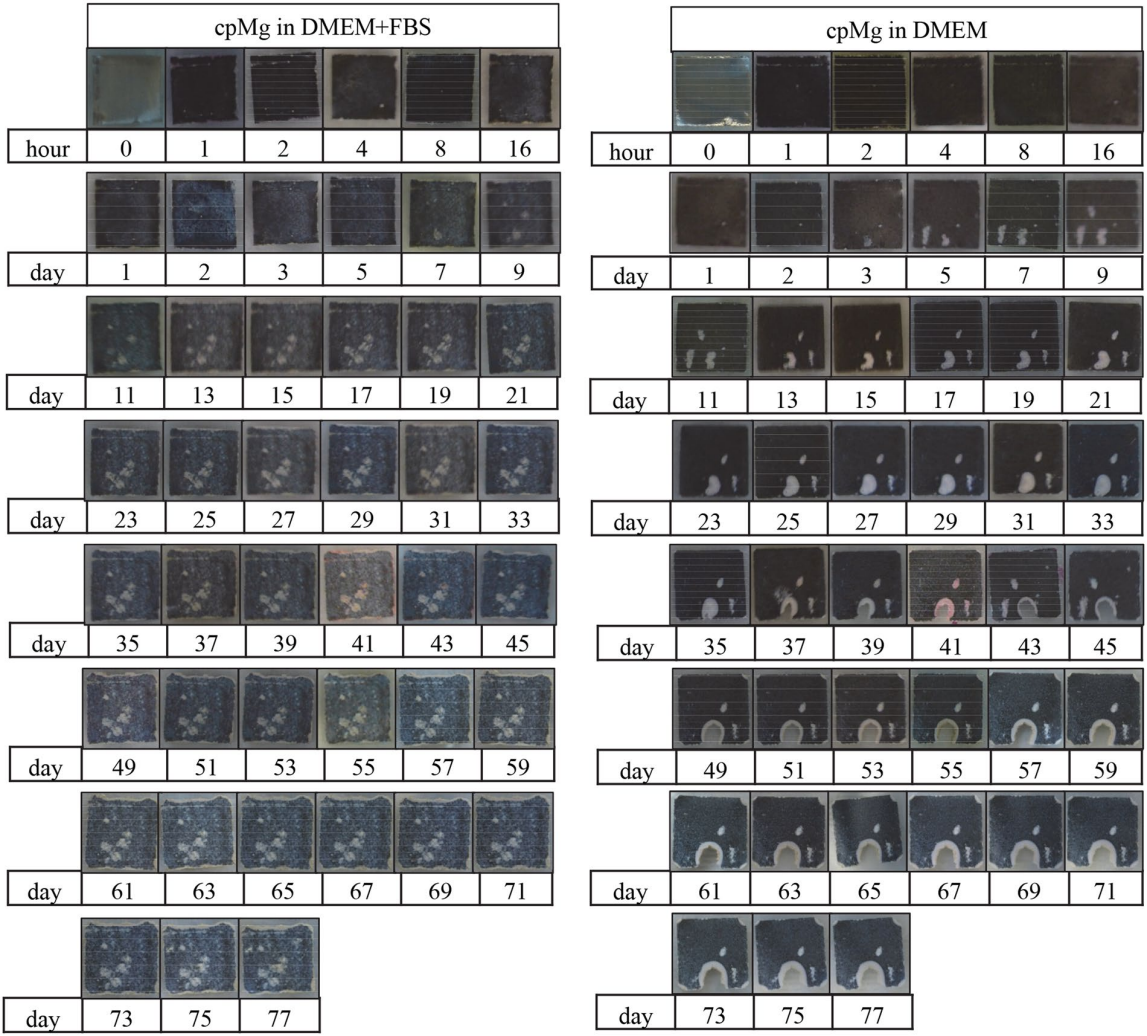
Photographs of cpMg showing its macroscopic appearance during immersion degradation in DMEM with and without FBS.

Immersion of the cpMg* samples in DMEM with or without FBS had significant effects on their surfaces (Figs 3 and 4). The entire surfaces of cpMg_O (Fig. 3) and cpMg (Fig. 4) darkened and acquired several semi-circular white deposits where there was significant localized degradation. The edges of cpMg* in DMEM with FBS receded inwards slightly. The appearance of cpMg* post-immersion in DMEM with and without FBS were initially similar, but eventually the growth of some of the semi-circular white deposits penetrated through the entire thickness of the samples when the immersion solution did not contain FBS; this created large holes that expanded laterally along the sample surface over time.

**Figure 5:**
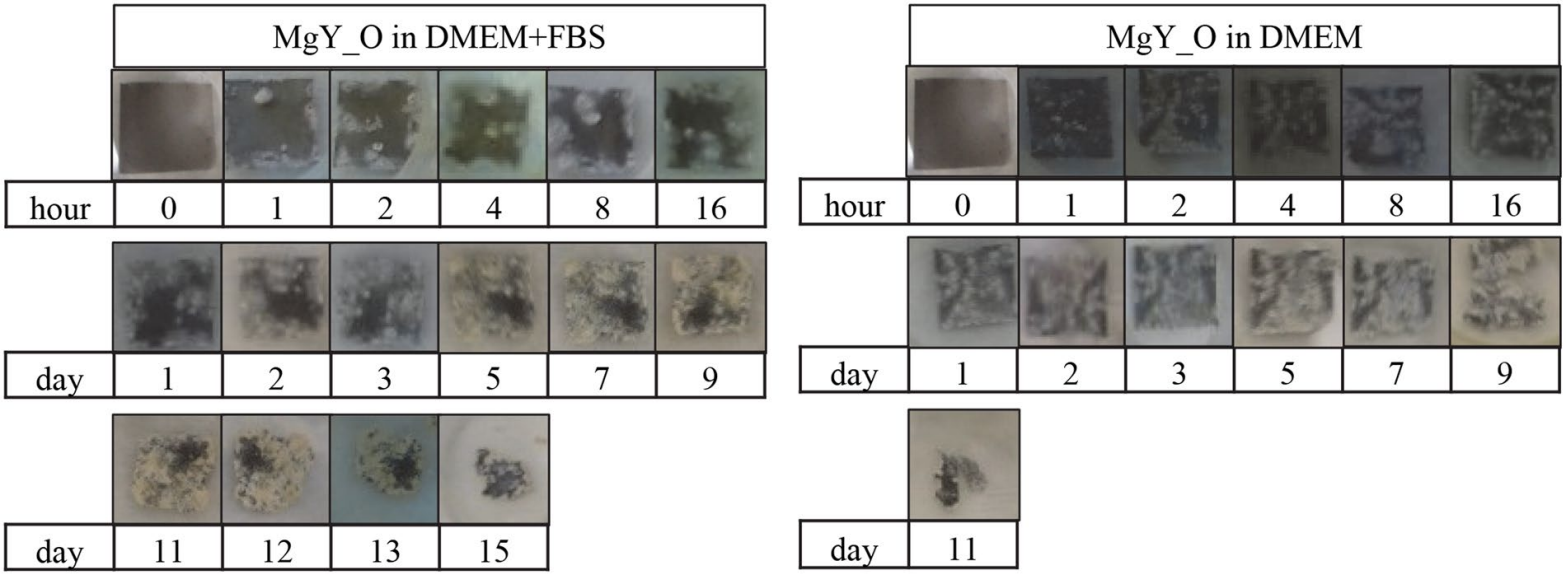
Photographs of MgY_O showing its macroscopic appearance during immersion degradation in DMEM with and without FBS.

**Figure 6:**
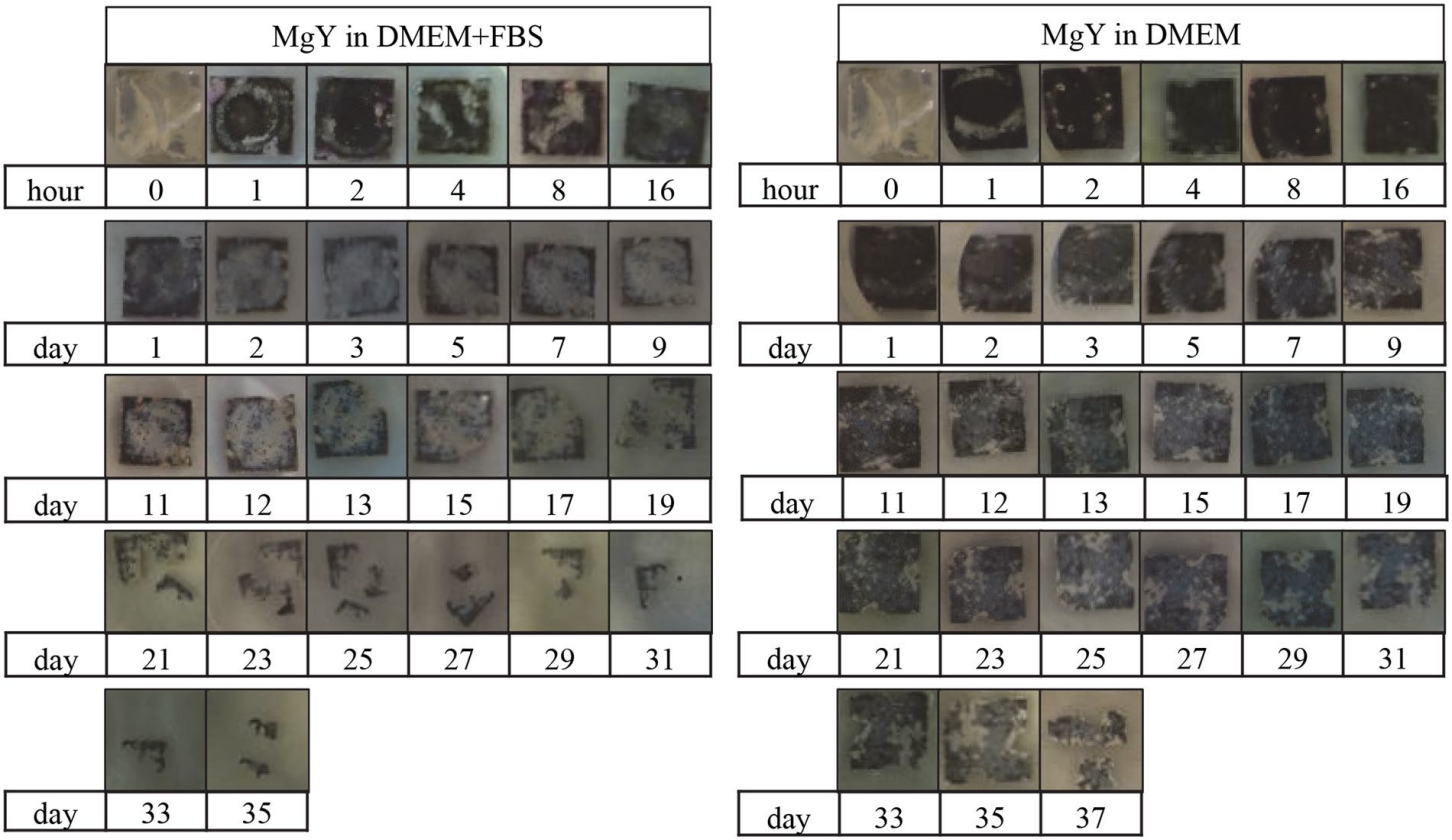
Photographs of MgY showing its macroscopic appearance during immersion degradation in DMEM with and without FBS.

Immersion of the MgY* samples in DMEM with or without FBS also showed significant effects on their surfaces (Figs 5 and 6). MgY* degraded much more quickly than cpMg*. The white deposits on MgY* were more numerous but smaller than those on cpMg*. FBS decreased the degradation rate of MgY_O, but conversely increased the degradation rate of MgY. In the presence of FBS, the edges of MgY_O receded inwards until no sample was left (Fig. 5). In the absence of FBS, however, MgY_O broke apart into several fragments near its center, and completely dissolved several days before its counterpart in the immersion solution supplemented with FBS. On the other hand, MgY broke apart into several fragments whether or not the immersion solution contained FBS, but the fragmentation occurred earlier if FBS was present (Fig. 6). In short, FBS decreased the degradation of MgY_O but accelerated the degradation of MgY.

### The Early Changes to Surface Microstructure and Elemental Composition

**Figure 7:**
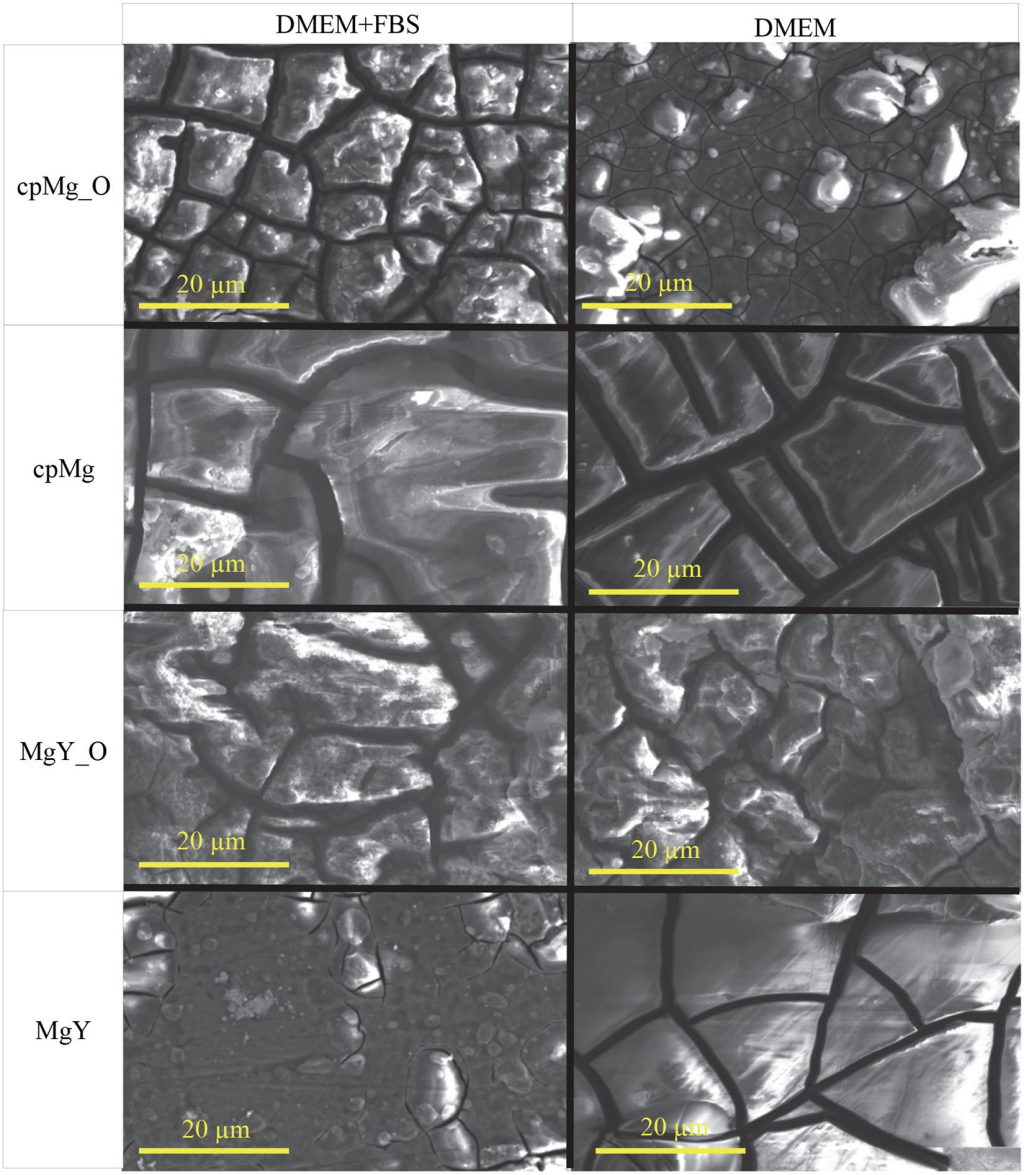
SEM images of cpMg* and MgY* after immersion in DMEM with or without FBS for 24 hours. Images were taken at an acceleration voltage of 15 kV with an original magnification of 2500X. Scale bars are 20 µm.

The cracks on the surface of cpMg_O after 24 hours of degradation appeared larger when the immersion solution contained FBS than when it did not (Fig. 7). The surface morphology of cpMg after 24 hours of degradation was nearly the same whether or not the immersion solution contained proteins. Proteins in the immersion solution had negligible effect on the surface morphology of MgY_O after 24 hours of degradation. However, FBS in the immersion solution reduced the cracks in the surface of MgY after 24 hours of degradation, which differed from what was observed with cpMg* and MgY_O.

**Figure 8:**
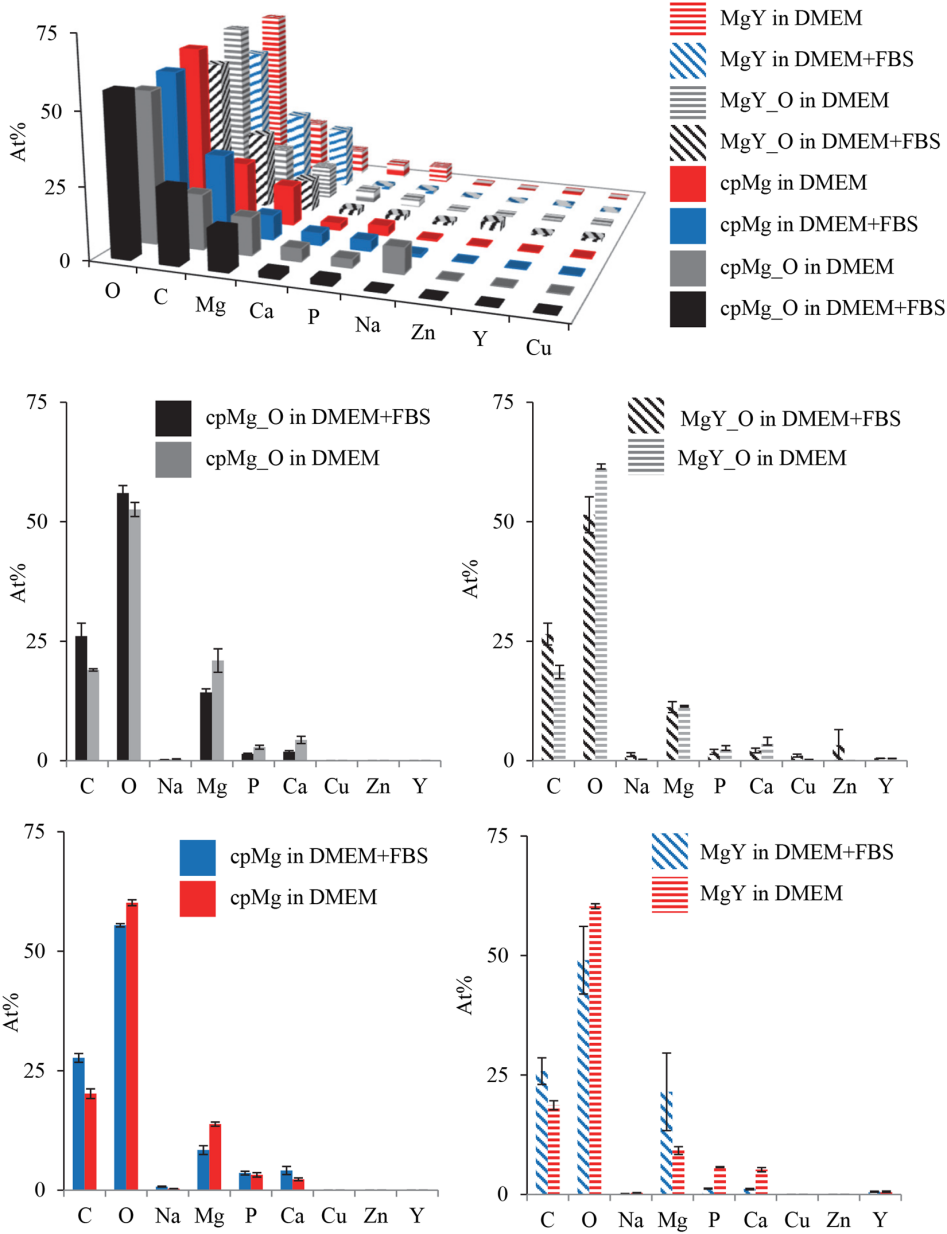
Surface elemental composition of cpMg* and MgY* after immersion in DMEM with or without FBS for 24 hours. The data were plotted in the 3D graph at the top for easy comparison and in four 2D graphs for visual clarity. Elements that were lower than 1 at % were not included. Elemental analyses were performed using EDX at an accelerating voltage of 15 kV. Error bars represent standard error. For each sample type in each 2D graph, no statistically significant difference was detected for elemental composition after immersion in DMEM with versus without FBS.

The presence or absence of FBS in the immersion media did not induce statistically detectable change on the surface elemental composition after 24 hours of immersion for cpMg* and MgY* (Fig. 8). Specifically, no statistically significant difference was detected for surface elemental composition of each sample type after immersion in DMEM in the presence versus absence of FBS, as shown in the 2D graphs in Fig. 8. Even though not statistically significant, on average, however, carbon (C) content on the surface always appeared higher on the samples immersed in the presence of FBS than the corresponding samples that were immersed in DMEM without FBS; FBS in the immersion solution resulted in less Mg on the surface for cpMg*, more Mg on the surface for MgY, and negligible difference for MgY_O, in close agreement with SEM observation in Fig. 7. The lack of statistical significance of FBS on the surface elemental composition might have partially been because the Bonferroni correction is overly conservative.

### Potentiodynamic Polarization Testing of cpMg* and MgY*

**Figure 9:**
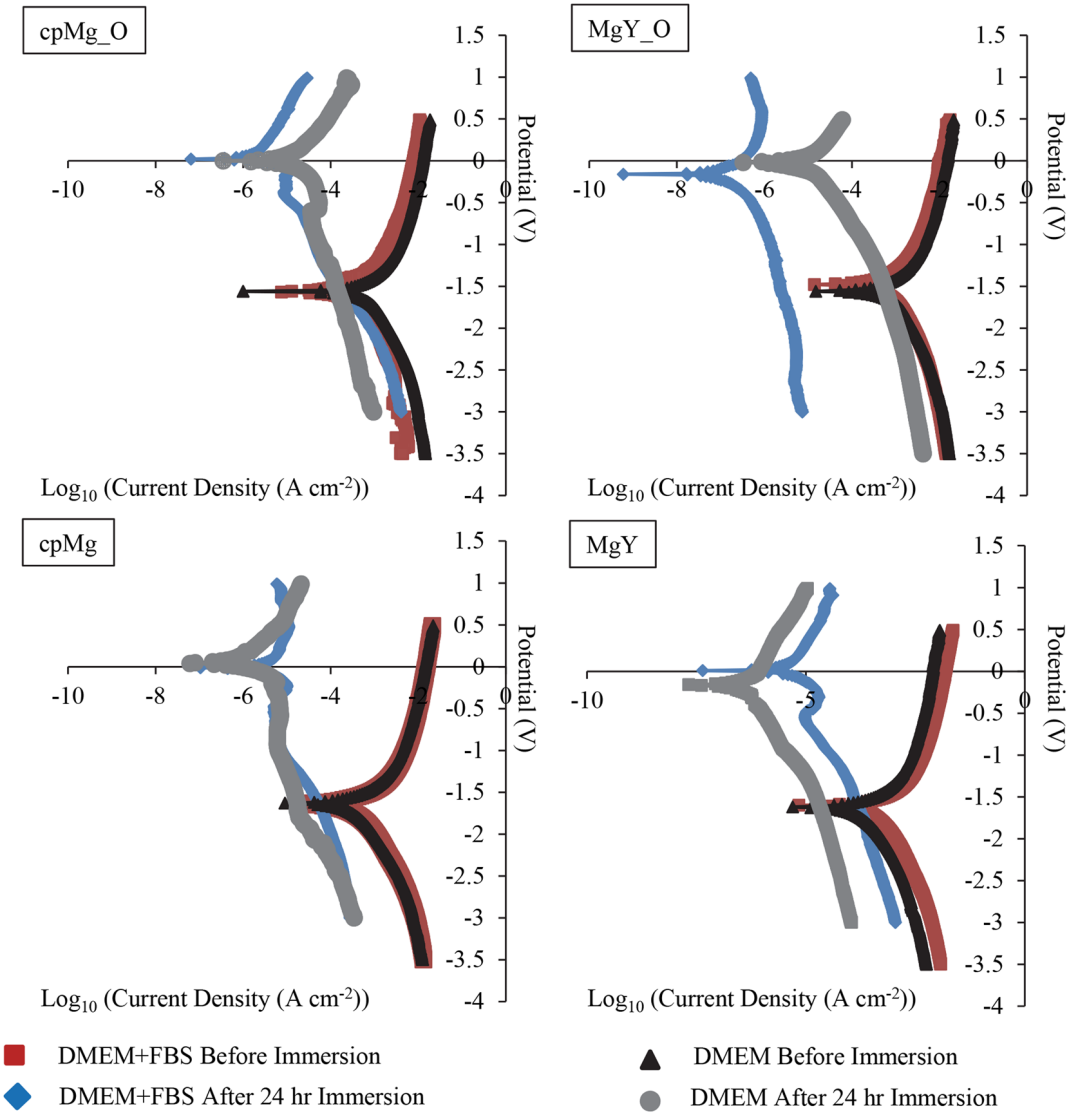
Representative potentiodynamic polarization curves of cpMg* and MgY* in DMEM with and without FBS, before and after 24 hours of immersion. All samples before immersion were cleaned and disinfected prior to the potentiodynamic testing.

**Figure 10:**
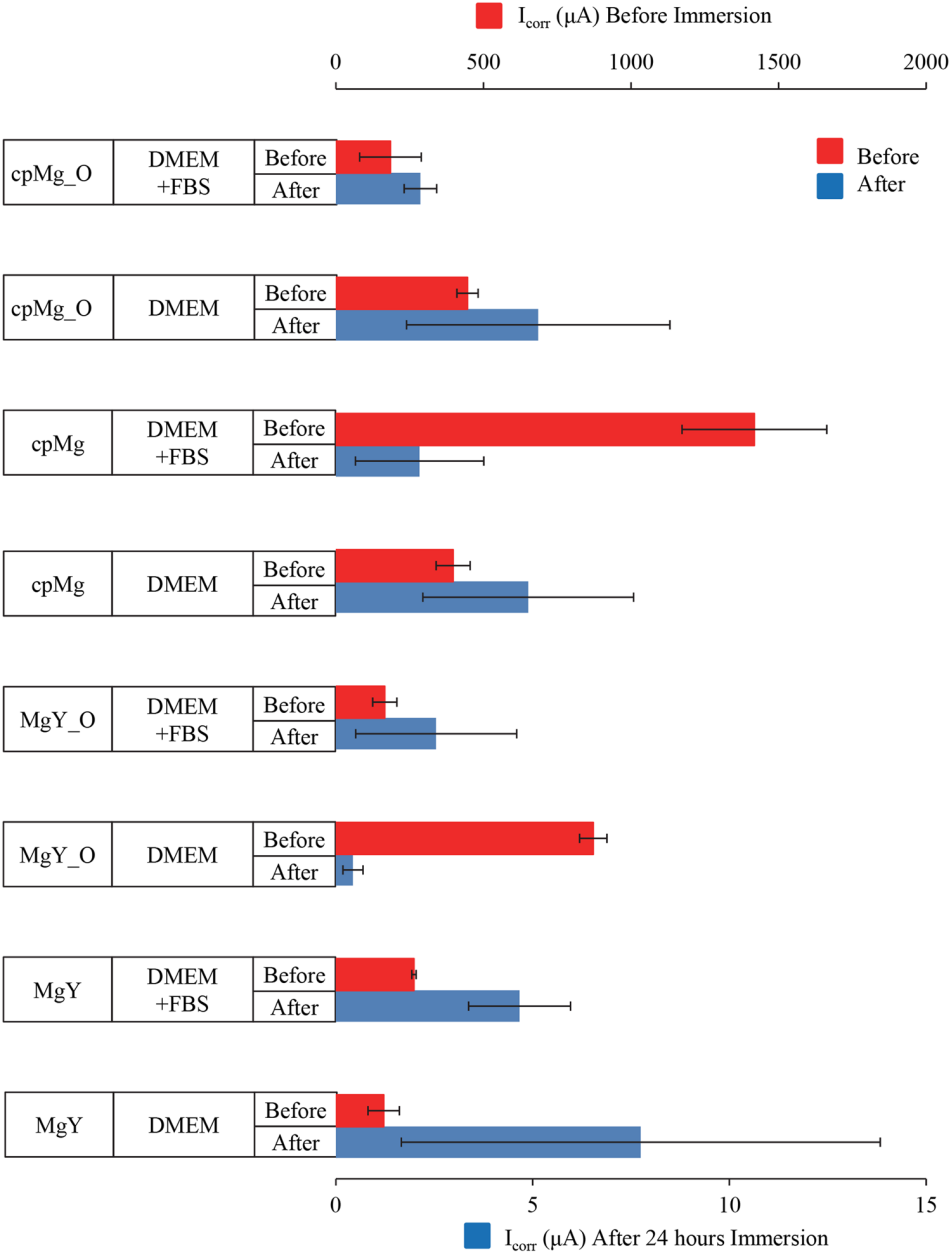
Corrosion current (I_corr_) extrapolated from potentiodynamic polarization curves of cpMg* and MgY* in DMEM with and without FBS. Measurements were made before and after 24 hours of immersion. Two different scales were used for I_corr_ values measured before (top scale) and after immersion (bottom scale). The error bars represent the standard error.

**Figure 11:**
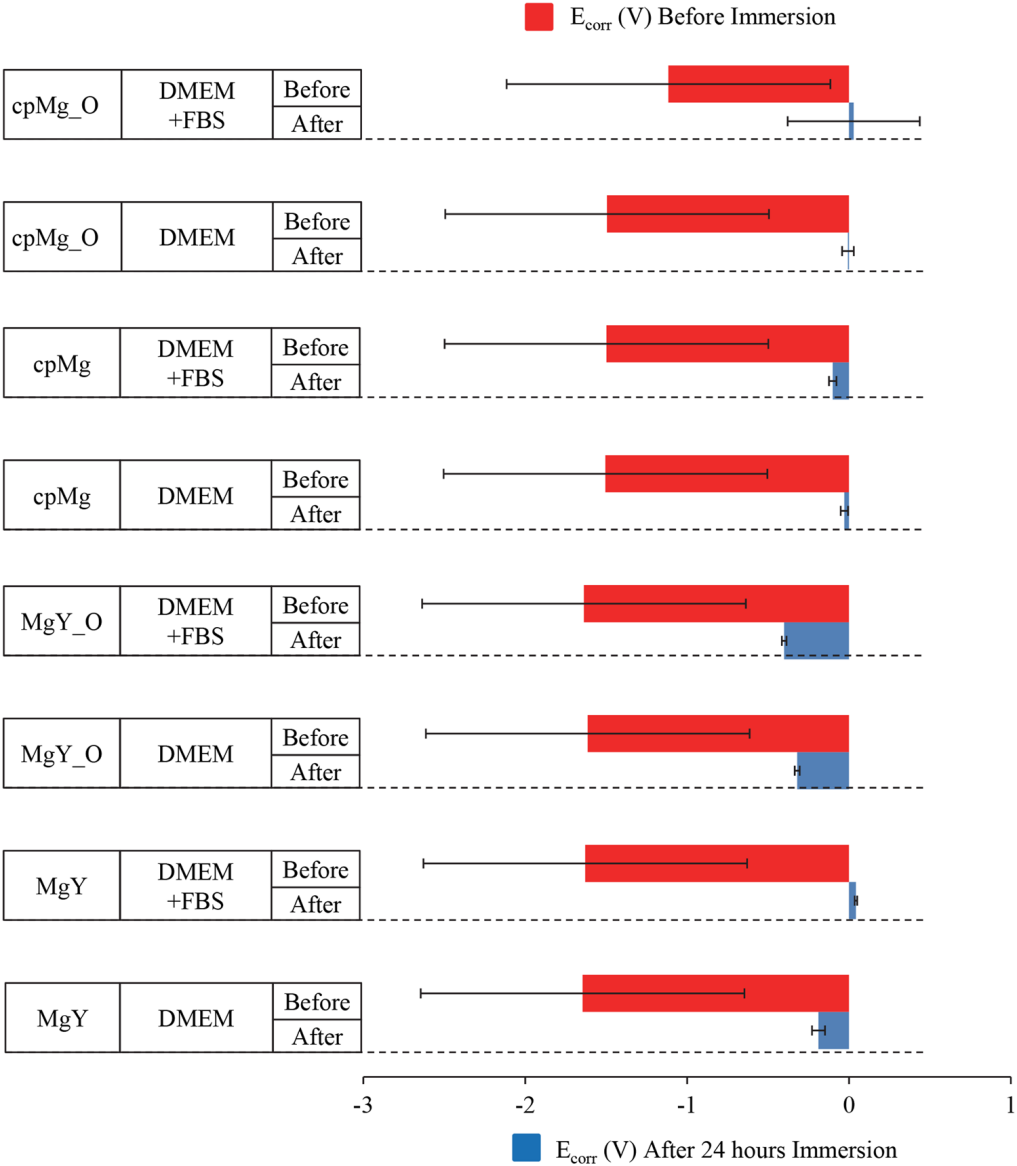
Corrosion potential (E_corr_) extrapolated from potentiodynamic polarization curves of cpMg* and MgY* in DMEM with and without FBS. Measurements were made before and after 24 hours of immersion. The error bars represent the standard error.

Immersion always reduced the corrosion current I_corr_ and increased the corrosion potential E_corr_ of all the samples (Figs 9–11). The potentiodynamic polarization curves before 24-hour immersion were symmetrical while the potentiodynamic polarization curves after 24-hour immersion were usually less symmetrical. After 24 hours of immersion, the anodic curves retained their usual shape, but the cathodic curves often deviated from symmetry near the corrosion potential. The most common examples of asymmetry for the cathodic curves were: (1) a nearly horizontal cathodic curve near the corrosion potential that eventually dropped sharply, and (2) a smaller second peak below the corrosion potential.

The presence of FBS in the immersion solution affected I_corr_ of cpMg* and MgY* (Fig. 10) and interacted with the other factors (Table 1). FBS by itself did not have any statistically significant effect on the I_corr_ of the samples (Table 1), although FBS did have statistically significant interactions with alloy composition (*p* = 2.39 × 10^−4^), with surface type (*p* = 7.99 × 10^−5^), with alloy composition and time (*p* = 3.59 × 10^−4^), and with surface type and time (*p* = 7.99 × 10^−5^). FBS reduced the I_corr_ of cpMg_O both before and after 24-hour immersion; but for cpMg FBS increased the I_corr_ before immersion and reduced the I_corr_ after immersion. FBS reduced the I_corr_ of MgY_O before immersion and then increased it after 24-hour immersion. FBS slightly increased the I_corr_ of MgY before immersion and reduced the I_corr_ after 24-hour immersion. FBS and its interactions did not have statistically significant effect on E_corr_.

## Discussions

### Proteins Affected Magnesium Degradation and Interacted with Other Factors

Proteins generally concentrate at the interfaces due to their amphoteric nature^13^, which could cause significant concentration of proteins at metal surfaces. The concentration of the adsorbed protein layer can come near the theoretical maximum density based upon the mass and volume of the proteins^4^. These proteins could promote the interactions with the metallic surface and oxide layer^4^. These interactions could be further promoted when the adsorbed proteins are partially desolvated at their interface with the metal surface^13^. The adsorbed protein layer is involved in many processes that could either inhibit or promote Mg degradation.

Protein adsorption on metal surfaces could create a physical barrier that protects the surface against electrolytes and aggressive ions^14^. Protein is also known to inhibit cathodic half-reactions by physically blocking cathodic sites^15^. Negatively charged proteins are more strongly attracted to cathodic sites on degrading metals, and can inhibit degradation by competing with Cl^−^ for adsorption^16^. The adsorbed protein barrier will not completely stop Mg degradation because the rigidity of protein chains limits how tightly they may be packed; which creates pores in the adsorbed protein layer that permit the transport of electrolyte and aggressive ions^13^. Electrons are able to travel through compact protein layers by making a series of discrete jumps through the proteins^17^, which permits degradation reactions to occur. Additionally, the adsorbed protein layer requires sufficient coverage and compactness so that the barrier properties of the protein layer outweigh the degradation promoting activities of proteins. A low concentration of albumin can lead to an incomplete protein layer that does not hinder the diffusion of aggressive ions and actually increases Mg degradation, while a higher concentration of albumin can create a more effective barrier that inhibits diffusion of aggressive ions and reduces Mg degradation^14^. Paradoxically, in some cases a protein barrier may actually promote degradation by limiting the formation of a stable degradation layer^18^.

Proteins undergo structural re-arrangements upon adsorption to a surface, some of these re-arrangements bring the hydrophobic interior of the protein in direct contact with the surface onto which they adsorbed^19^. This brings the internal disulfide bridges of globular proteins (e.g. albumin) into direct contact with metal surfaces where they can catalyze metal oxidation^20,21^. The disulfide bridges can be reduced by the metal surface and then be re-oxidized by oxygen^20^, which enables repeated reactions between the disulfide bridges and the metal surface.

The I_corr_ value extrapolated from the potentiodynamic polarization curves (Fig. 10) is an important indicator for predicting metal corrosion, but it does not always agree with the degradation results from immersion study. It is important to note the different time scales over which the potentiodynamic polarization and immersion tests were performed. The potentiodynamic polarization tests were far too short to account for all the different effects of proteins during the entire immersion. Similar time-scale effect was also observed in other literatures^22–24^. After 24 hours of immersion, the edges of the MgY_O samples were covered with numerous large white deposits that proliferated inwards and coalesced with each other over time, which undermined the integrity of the MgY_O surface oxide layer. On the other hand, FBS caused MgY samples to form a white deposit film on most of the surface, but with less deposition at the edges. The differences in the deposition patterns of precipitates on MgY_O and MgY were due to several reasons. The faces of the MgY_O samples had the initial thermal oxide layer, but the edges were cut with scissors and thus were metallic surfaces. The edges were the most rapidly degrading part of the samples and had the greatest electrostatic attraction for negatively charged proteins, which are abundant in the serum. Conversely, the faces and edges of the MgY samples all had similar metallic surfaces initially. The greatest electrostatic attraction for proteins may have been spread across the samples faces instead of concentrated along the edges, which led to the white deposit film appearing on the center of the sample faces. The influence of FBS on the white deposits was more apparent in MgY* than cpMg* because proteins tend to have more pronounced effect on more rapidly degrading alloys.

The growth of the white deposit layer on MgY in the presence of FBS corresponded to increased degradation during the immersion study. However, this layer of deposits reduced I_corr_ after 24 hours of immersion because it was a barrier to diffusion during the short duration of potentiodynamic polarization testing, which was also demonstrated by the smaller size of the surface cracks on MgY after 24 hours of immersion when FBS was present in the immersion media (Fig. 7).

### Alloy Composition and Processing Modified the Interactions between Magnesium Surfaces and Proteins

Proteins showed different effects on the degradation of cpMg* and MgY* because of the competing processes that the proteins participated in^13–16^, the diversity of the proteins in FBS which may be preferentially adsorbed onto certain surfaces^20^, the composition of the Mg alloys, and the surface properties of the Mg substrates. The effects of proteins in DMEM on the degradation properties of cpMg* and MgY* are summarized in Table 2. In general, proteins have less effect on the degradation of well passivated, slower degrading alloys^20^. Our results showed that the presence of FBS had only minor effect on mass changes of cpMg* (Fig. 1). Considering the high purity and uniform microstructure of cpMg* samples, only a few large semi-circular white deposits formed on the surface and the degradation of cpMg* was localized on the few present faults or impurities (Figs 3 and 4). Consequently, cpMg* degraded more slowly than the other groups. Although FBS did not have significant effects on mass changes of the samples, it did alter the degradation mode. The presence of FBS in the immersion solution prevented pitting degradation from penetrating through the entire thickness of the cpMg* substrates and spreading laterally to form large holes as observed with cpMg* in the absence of FBS. The adsorbed proteins might have provided a physical barrier that interfered with aggressive ions from attacking the surface.

**Table 2:** A summary of the effects of proteins in DMEM on the degradation properties of cpMg* and MgY*.

The Effects of Proteins on Magnesium Alloy Degradation			
Sample	Immersion Study		Electrochemical Test Before/After 24-hr Immersion
	Mass Changes	Visual Changes	I_corr_
cpMg_O	Inhibited mass loss slightly after 40 days	Similar degradation rate, but prevented full penetration through sample	Reduced before and after immersion
cpMg	Negligible		Increased before immersion but reduced after immersion
MgY_O	Inhibited mass loss	Decreased degradation and prevented fragmentation	Reduced before immersion and increased after immersion
MgY	Greatly promoted mass loss	Promoted degradation and fragmentation	Increased before immersion and reduced after immersion

In comparison with cpMg*, the MgY* samples showed larger and more rapid mass loss (Fig. 1). We observed that small white deposits on the MgY* samples were numerous and dispersed across the entire surface after immersion; and that these deposits proliferated and merged with each other until they covered most of the surface area (Figs 5 and 6). The Mg and Y phases of Mg-Y binary alloys often form galvanic couples^25^, which could create an abundance of localized corrosion cells at the surface of the samples^26^. The presence of FBS in the immersion solution had significant effects on MgY* degradation, because proteins tend to have greater influence on more rapidly degrading metals^20^. The negatively charged albumin was more strongly attracted to the anodic regions of the samples where degradation was most rapid. The adsorbed proteins exerted both degradation inhibiting and promoting effects; our results showed that protein had a net inhibiting effect on MgY_O degradation, but a net promoting effect on MgY degradation. FBS decreased the degradation of MgY_O by preventing pitting degradation processes from penetrating all the way through the MgY_O substrates, which reduced sample fragmentation during immersion degradation; and was similar to the effects of FBS on cpMg* degradation.

In contrast, the degradation rate of MgY increased with the presence of FBS. MgY did not have the initial oxide layer that MgY_O did, and thus its entire oxide layer was formed in the presence of FBS. The extraction of metal ions and oxides and the inhibition of phosphate incorporation prevented the formation of a stable and protective oxide layer on MgY. Additionally, the proteins in FBS could adsorb directly to metallic surface of MgY, without an oxide layer between the proteins and the metallic surface. The disulfide groups of proteins could catalyze MgY degradation more easily when there was no oxide barrier between them and the metallic surface. Proteins are known to have greater influence on more rapidly degrading alloys^20^, which may explain why the FBS had more significant effect on mass loss of MgY during immersion than on mass loss of cpMg. The complex and often competing effects of proteins on Mg degradation require further research to improve our ability to control the degradation of Mg-based biomaterials.

## Conclusions

Proteins engaged in multiple processes that could potentially inhibit or promote Mg degradation, depending on the type of proteins and the properties of the Mg surface. FBS inhibited degradation of cpMg_O, cpMg, and MgY_O by limiting the depth of pit formation; which reduced sample fragmentation. In contrast, FBS promoted degradation of MgY, which may have been caused by chelation of metal ions and oxides or promotion of local galvanic cells. The results of this study demonstrated that serum proteins had significant interactions with Mg-based medical implants and devices, which may be modified by the composition and processing of Mg alloys. Proteins should be taken into account when designing *in vitro* experiments to determine degradation of Mg-based implants.
